# MEASURING EFFECT OF PERSONALIZED MUSIC TO REDUCE BEHAVIORS IN NURSING HOME RESIDENTS WITH DEMENTIA

**DOI:** 10.1093/geroni/igz038.205

**Published:** 2019-11-08

**Authors:** Ellen McCreedy, Xiaofei Yang, Rosa Baier, Kali S Thomas, James Rudolph, Vincent Mor

**Affiliations:** 1 Brown University, School of Public Health, Providence, Rhode Island, United States; 2 Brown University, Providence, Rhode Island, United States; 3 Providence VA Medical Center, Providence, Rhode Island, United States; 4 Providence VA Medical Center, Center of Innovation in Long Term Services and Supports, Providence, Rhode Island, United States

## Abstract

The purpose of this six-month pilot study was to identify an optimal measurement strategy for assessing the effects of a personalized music program, MUSIC & MEMORY, on agitated and aggressive behaviors among 45 nursing home residents with moderate to severe dementia. Dementia-related behaviors were measured before and after the intervention with three methods 1) observationally using the Agitation Behavior Mapping Instrument (ABMI); 2) staff report using the Cohen-Mansfield Agitation Inventory (CMAI); and 3) administratively using Minimum Data Set - Aggressive Behavior Scale (MDS-ABS). ABMI score was 4.4 (standard deviation, SD: 2.3) while not listening to the music and 1.6 (SD: 1.5) while listening to music (p<.01). CMAI score was 61.24 (SD: 16.32) before the music and 51.24 (SD: 16.05) after the music (p<.01). MDS-ABS score was .8 (SD: 1.6) before music and .7 (SD: 1.4) after music (p=.59). Direct observations were most likely to capture behavioral responses, followed by staff interviews. No effect was found using exclusively available administrative data. There is growing interest in identifying and testing non-pharmaceutical alternatives to managing agitated and aggressive behaviors in nursing home residents with dementia. Measurement occurring closest in time to the intervention was most likely to capture responses, but was also most costly, least pragmatic, and most subject to confirmation bias. These findings will inform a large pragmatic trial, beginning Spring 2019.

